# Early experience of multi-sequence fetal cardiac magnetic resonance imaging within a clinical fetal cardiology service

**DOI:** 10.1186/1532-429X-18-S1-P177

**Published:** 2016-01-27

**Authors:** David Lloyd, Joshua F van Amerom, Kuberan Pushparajah, John M Simpson, Vita Zidere, Owen Miller, Gurleen Sharland, Joanna Allsop, Matthew Fox, Christina Malamateniou, Joseph V Hajnal, Mary Rutherford, Reza Razavi

**Affiliations:** 1 Evelina Children's Hospital, London, United Kingdom; 2 King's College London, London, United Kingdom

## Background

Prenatal diagnosis of congenital heart disease is important to ensure both informed parental counselling before birth, and effective planning of potentially life-saving interventions after birth. MRI offers a safe, radiation-free adjunct to conventional diagnostic modalities, particularly in mothers with high BMI or at later gestations where ultrasound may be limited. However, cardiovascular imaging in the fetus presents several challenges, not least due to the small size and constant motion of the fetal heart, the lack of external cardiac gating, and gross fetal and maternal motion. We present our initial experience of 20 fetal cardiac MRI cases, each referred following fetal echocardiographic assessment to resolve specific points of diagnostic uncertainty.

## Methods

Referrals were based on the judgements of the attending fetal cardiologists between June 2014 and May 2015. A paediatric cardiologist with expertise in cardiac MR was present at every scan. Following a three-plan localiser, diagnostic sequences were generally limited to half-Fourier acquisition single-shot turbo spin-echo (HASTE) and balanced steady state free precession (bSSFP) gradient echo sequences. A large field of view bSSFP cine (real-time) scan with low spatial resolution but higher temporal resolution (303 ms) was used following the localiser to demonstrate the degree of gross fetal movement. If needed, further shortened localiser sequences were also used, followed by bSSFP scans with higher spatial resolution for diagnostic purposes.

## Results

Single-shot turbo spin-echo (HASTE) sequences produced T2 weighted images with black-blood like contrast which was particularly useful for assessing the extracardiac vascular anatomy. Balanced SSFP images gave good contrast between the blood pool and surrounding tissue and were useful for intracardiac structures; however these were more susceptible to motion artefacts. Real-time SSFP sequences allowed for dynamic imaging of the beating heart in assessing moving structures (e.g. cardiac masses and diverticulums); when used in combination with HASTE sequences, we were able to characterise rhabdomyomas in three patients.

## Conclusions

Our preliminary experience of fetal cardiac MRI suggests that it can provide safe, clinically useful complimentary imaging in the majority of selected cases within a tertiary fetal cardiac unit, particularly for extracardiac vascular anatomy and intracardiac masses. In the future, prenatal MRI may have a more prominent role in routine fetal cardiovascular assessment.

**Figure 1:**
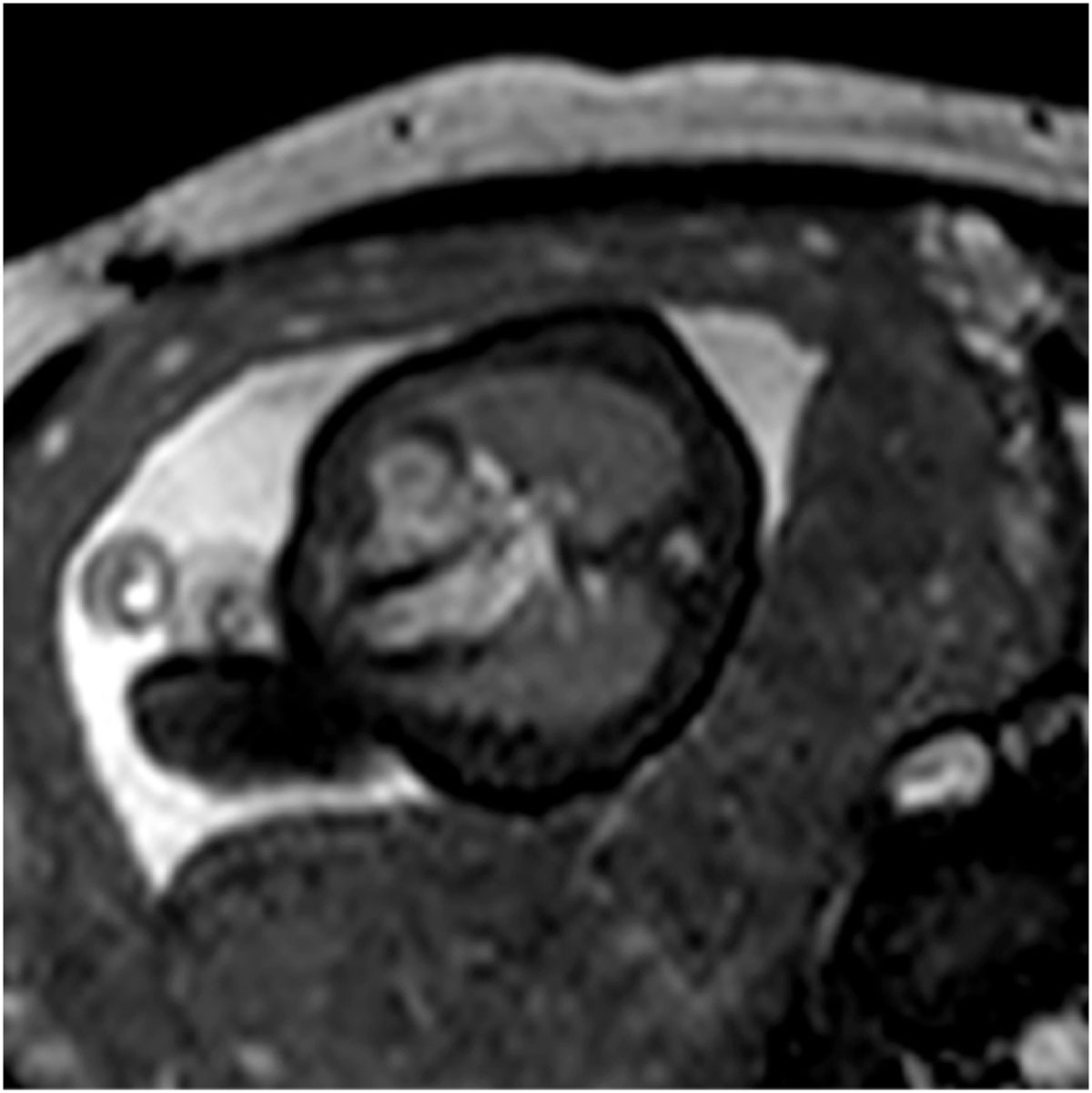
**Balanced SSFP bright-blood four chamber view of the heart in a 30+3 fetus showing a diverticulum of the right ventricle**.

**Figure 2:**
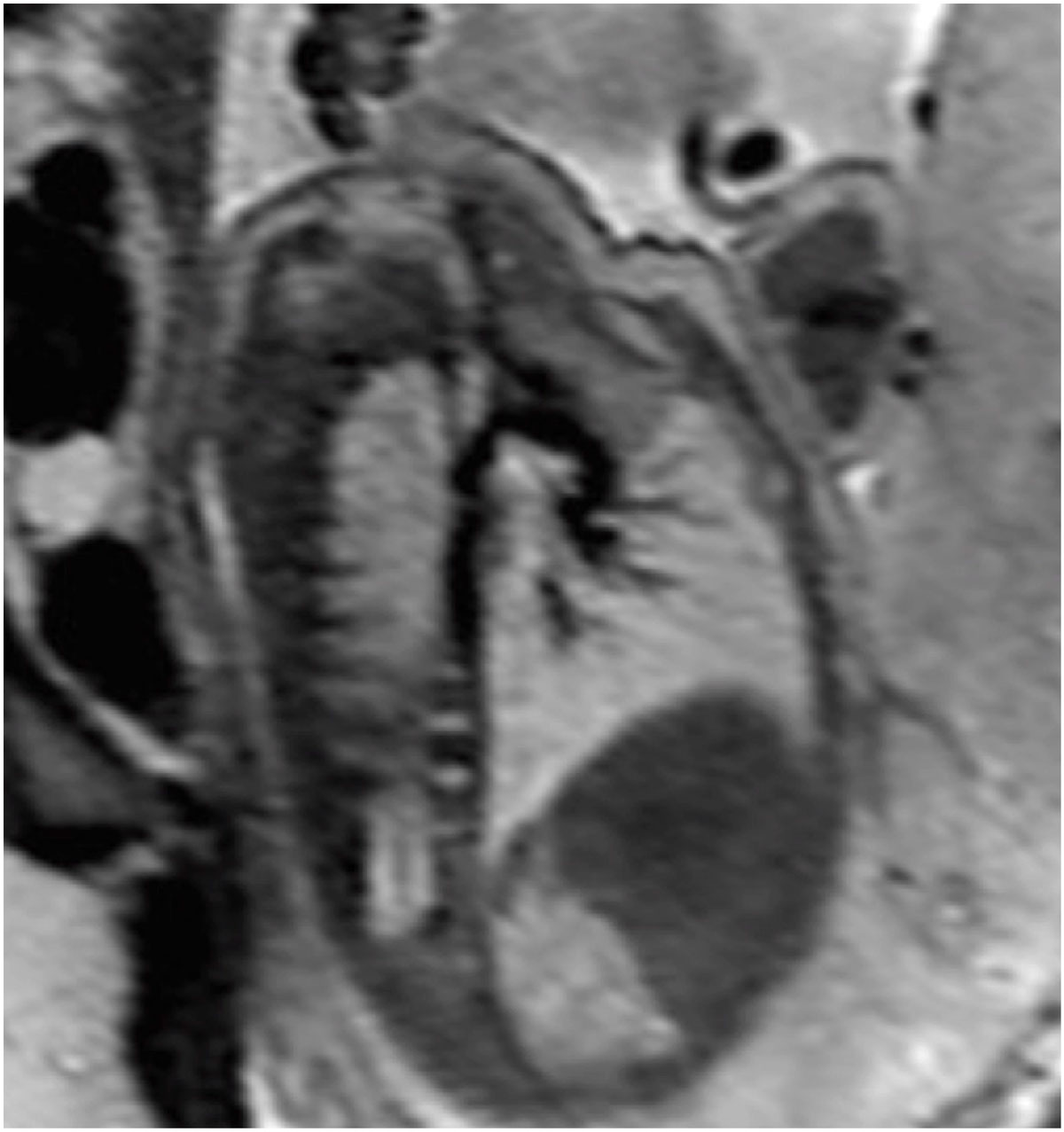
**Oblique single-shot fast spin echo black-blood image of the aortic arch in a 32+2 week fetus with coarctation of the aorta**.

